# Investigating experiential effects in online chess using a hierarchical Bayesian analysis

**DOI:** 10.1515/jqas-2025-0035

**Published:** 2025-09-19

**Authors:** Adam Gee, Sydney O. Seese, James P. Curley, Owen G. Ward

**Affiliations:** Department of Statistics and Actuarial Science, Simon Fraser University, Burnaby, BC, Canada; Department of Psychology, The University of Texas at Austin, Austin, TX, USA

**Keywords:** winner-loser effects, chess, hierarchical Bayesian modeling, online competitions

## Abstract

The presence or absence of winner-loser effects is a widely discussed phenomenon across both sports and psychology research. Investigation of such effects is often hampered by the limited availability of data. Online chess has exploded in popularity in recent years and provides vast amounts of data which can be used to explore this question. With a hierarchical Bayesian regression model, we carefully investigate the presence of such experiential effects in online chess. Using a large quantity of online chess data, we see little evidence for experiential effects that are consistent across all players, with some individual players showing some evidence for such effects. Given the challenging temporal nature of this data, we discuss several methods for assessing the suitability of our model and carefully check its validity.

## 1 Introduction

It is rare that the success or failure of an individual competitor or sports team is not discussed in the context of their recent performance. The idea of momentum in competition, broadly defined, has been examined repeatedly in the scientific literature. In sports analytics this is often investigated in the context of the “hot hand” phenomenon, where players experience streaks of success that appear unlikely to occur due to chance (Miller and Sanjurjo 2018; Ötting et al. 2020; Pelechrinis and Winston 2022). Similarly, other studies have directly considered the question of momentum in sport, both within and between games (Gauriot and Page 2018; Steeger et al. 2021).

A large body of research in the psychology and animal behavior literature explores a specific formulation of this concept of momentum. It is commonly referred to as winner-loser or experiential effects,1We will use these two names interchangeably throughout this work. defined as success (or failure) in recent competition leading to an increased probability of success (or failure) in future competition (Dugatkin 1997). The presence of these winner-loser effects have been hypothesized to facilitate the formation of stable dominance hierarchies (Dugatkin 1997; Kura et al. 2015). In animals, loser effects appear to consistently be stronger than winner effects (e.g. sticklebacks (Bakker 1986); crayfish (Bergman and Moore 2003); copperhead snakes (Schuett 1997)). In humans, several studies have revealed some evidence for winner and loser effects in competitive sports including tennis, football and judo (Cohen-Zada et al. 2017b; Gauriot and Page 2018; Gauriot and Page 2019; Page and Coates 2017). For example, winners of tennis tie-breaks are more likely to win subsequent sets when players are evenly matched (Page and Coates 2017). Winner and loser effects have also been experimentally demonstrated in humans playing competitive video games (Smith and Dukas 2024). However, there is still considerable discussion about the existence and consistency of these effects across individuals, contexts and species.

Indeed, there are still several outstanding key questions regarding the presence of such experiential effects, including

– Are winner/loser effects strongest when considering the immediate previous outcome or several previous outcomes?
– Do the size of winner/loser effects vary across rating levels such as between players/teams of stronger versus lesser ability?
– Are winner/loser effects stable across time?
– Do winner/loser effects vary within individuals across contexts such as across different formats of competitions?

One of the main challenges posed by these questions is the availability of appropriate data to investigate these phenomena. Online chess has enjoyed widespread popularity in recent years, with millions of regularly active users on several major sites. The most common format of chess played on these sites is “Standard” rated games. These games are often short “bullet” games, lasting less than 3 min. It is common for players to play many such games in succession. In these games, players are randomly paired with another user currently wishing to play at the same time and of similar estimated ability. The color that each player is assigned (White or Black) is randomly chosen. Games of this form provide an excellent setting to investigate the presence of psychological momentum and experiential effects in such sports and can be used to consider the key questions posed above. Any individual plays a wide range of players, who may utilise different styles and strategies of chess. The random assignment of opponents and individual game settings, coupled with the vast amount of games many users play, makes online chess an excellent setting to investigate the presence of winner-loser effects in a controlled environment.

In this paper we use a Bayesian hierarchical logistic regression model to investigate the presence of winner-loser effects in online chess, using publicly available data. We carefully motivate and describe the model chosen. Then, we use this model to examine if and when these effects occur, and how they vary across player ability and game format. While we do not believe this model is a complete representation of the process underlying these games, we use multiple model checking procedures to ensure our model is reasonable.

We find little evidence for strong persistent winner-loser effects, with this conclusion consistent across the range of players and abilities analysed. Individuals appear to show some variability, and there is some evidence that some players demonstrate such effects. While there is somewhat increased evidence for these winner-loser effects in the top international competitors, these effects do not appear to be systematic across the population of chess players analysed.

This article is organized as follows. We first provide a brief history of the study of these effects across disciplines in Section 1.1. In Section 2 we describe in detail the data we use here, highlighting key properties which make it well suited for the study of winner-loser effects. Section 3 gives the proposed hierarchical Bayesian regression model, detailing the model and prior specification. In Section 4 we fit the proposed model, highlighting the results for two cohorts of chess players of differing abilities. Section 5 provides a detailed discussion of one natural procedure used to ensure our model is able to correctly capture the temporal structure present in such data. We include several further model checking approaches in the Appendix. Finally, in Section 6 we summarise the results of this model and highlight important considerations for future work.

### 1.1 A history of the study of experiential effects in human and animal competition

Winner-loser effects have consistently been demonstrated across species engaged in various competitive interactions (Abe et al. 2021; Bakker and Sevenster 1983; Dugatkin and Druen 2004; Hsu and Wolf 1999; Kasumovic et al. 2010; Lan and Hsu 2011; Lehner et al. 2011; Lerena et al. 2021; Oldham et al. 2020; Oliveira et al. 2009; Oliveira et al. 2011; Schwartzer et al. 2013; Stevenson and Rillich 2013; Trannoy and Kravitz 2017). The most common experimental design utilizes a series of dyadic matchups whereby individuals are induced to win or lose against weaker or stronger opponents and are then evaluated in subsequent competitions. These studies have demonstrated that loser effects are typically more long lasting than winner effects (Abe et al. 2021; Lan and Hsu 2011; Lehner et al. 2011; Stevenson and Rillich 2013; Trannoy and Kravitz 2017). The likelihood of winning subsequent encounters has also been shown to increase following unresolved aggressive encounters (e.g. East African cichlid fish (Dijkstra et al. 2012)) or even following recent losses (olive fruit fly (Benelli et al. 2015)). Further, variation in competitive contexts or individual variability in skill level of fighting (Briffa and Lane 2017), growth rate (Li et al. 2023), age (Fawcett and Johnstone 2010), and life experience with fighting (Laskowski et al. 2016) can all influence the strength of winner-loser effects. Functionally, loser effects may serve to reduce aggression intensity within groups, with fewer attacks received by previous losers, resulting in fewer injuries to losers (Lehner et al. 2011; Stevenson and Rillich 2013). Winner effects serve to protect dominants from excessive energy usage by resolving aggressive encounters more efficiently (Lehner et al. 2011). Several theoretical models have demonstrated that winner-loser effects are sufficient to lead to the formation of stable dominance hierarchies (Dugatkin 1997; Dugatkin and Earley 2004).

There exists relatively less evidence for the presence of winner-loser effects in human competitive interactions despite their existence being strongly hypothesized. In humans, these effects are also referred to as psychological momentum which is defined as a change in the probability of an outcome occurring as a function of the outcome of preceding events (Adler 1981). For example, it has been reported that male judoists are more likely to win their next contest if they won their previous event (Cohen-Zada et al. 2017a), and male tennis players are more likely to win the next point if they won the preceding point (Gauriot and Page 2019). Male tennis players are also more likely to win a subsequent set against an evenly matched opponent after winning a close tiebreak (Page and Coates 2017). These effects were not observed in female players. Similar instances of psychological momentum have been observed from large datasets of competitive sports, including that hitting the bullseye in elite archery improves performance on the next shot (Zhao and Zhang 2023), and that the likelihood of winning double-headers in softball and baseball increases if both games are played on the same day rather than over multiple days (Eldakar et al. 2022; Gallup et al. 2018). Alternatively, researchers have experimentally manipulated the outcomes of competitive interactions in video games and shown that individuals who are randomly assigned to be winners perform better in future contests than do those who are randomly assigned to be losers (Smith and Dukas 2024).

Notably, there has been a relative lack of investigation of human competitive interactions that incorporate thousands of competitive interactions over a longer period of time such as is available with data from online chess matches. Similarly, the existing models for examining such effects are often limited or somewhat domain specific. While models such as Lerena et al. (2021) examine experiential effects in animals, their approach is largely exploratory, examining the relationship between several potential outcomes of interest and previous performance using correlation and generalized linear mixed models. This form of approach, along with traditional statistical tests, is common in the animal literature. In sports, models which have been proposed cannot easily be modified to other scenarios. While an advanced statistical model was proposed to investigate the hot hand in professional darts (Ötting et al. 2020), this utilises the fact that each player has three throws in each turn, examining experiential effects both within a turn and across a leg (several turns). It is unclear how to extend this to more general forms of competition or interactions. Similarly, Gauriot and Page (2018) use causal inference techniques to examine the impact of teams scoring before half time in soccer, matching these goals to similar attempts which instead hit the post. However, it may be challenging to modify this matching approach to other games or scenarios. In this work we develop a natural statistical model for such interaction data which can be applied to any dataset of repeated competition. As discussed below, it can also incorporate information about these competitions as available.

## 2 Data

**Figure 1:**
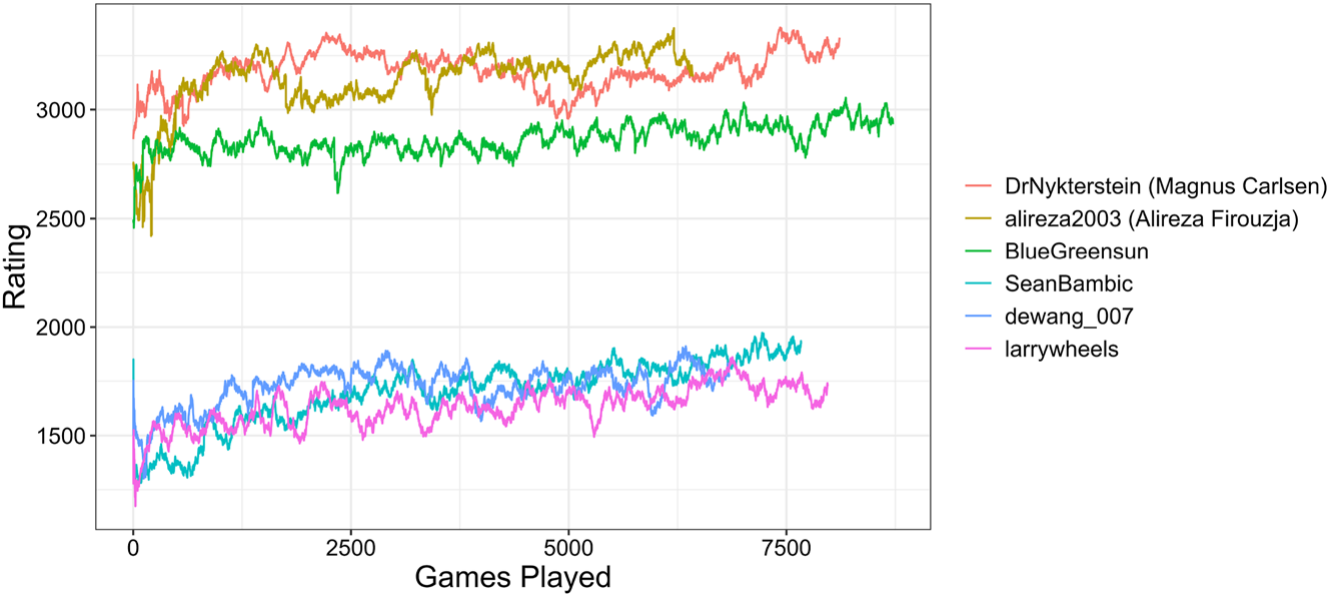
Evolution of bullet Glicko-2 ratings for 3 players in the GM cohort and 3 players in the 1700–1900 cohort. “DrNykterstein” (Magnus Carlsen) and “alireza2003” (Alireza Firouzja) are among the current top players in the world, with Carlsen being at the number 1 spot since 2011.

In this work we use rated standard games played on Lichess, a popular open source chess server. Lichess and other chess platforms pair random opponents who are believed to have a similar chess ability, to create games where both players have an opportunity to win. In online and over the board chess, it is common to estimate the ability of an individual using a numeric rating based on their past performance. When an individual creates an account on Lichess, they are a given a default rating of 1,500 across all chess formats. As the player plays rated games at a specific time format, their rating will update based on their performance and the rating of their opponents. Lichess uses the Glicko-2 rating system (Glickman 2001; Glickman and Jones 2024), which can also estimate a measure of variability of this rating. This new system builds on the original system of Glickman (1999) by producing larger variability in the estimated ratings if unexpected results occur. In Figure 1 we show the rating evolution of six players with a similar number of games played from the two cohorts we analyse in detail below. We include three players who are among the highest rated on Lichess (two of whom are known elite players). We also include three players of a more moderate ability. These players show some of the typical behaviour across the range of players we consider. These ratings can show large changes initially, with smaller changes present after several hundred games are played. Similarly, some of these players, such as “SeanBambic”, can show a gradual upward trend, indicating gradual improvement in skill over time.

Lichess allows a wide range of variants and time formats of chess, with games ranging from several minutes to several days. The only variants we consider are standard chess formats, which is the vast majority of games on Lichess. In these time controlled chess games, each player starts with the same amount of time to make their moves. The two most common formats are “Bullet” (games where each player has 1–2 min) and “Blitz” (games where each player has 3–5 min). These short games commonly end with one player winning via checkmate, resignation or running out of time. Draws are possible but relatively rare in these formats. We consider only the most played time controls in bullet and blitz for analysis: 60+0 and 180+0. The first number in these time controls represents how many seconds each player starts with to make all their moves. The second represents the increment, which is the number of additional seconds added to their remaining time after making each move.

We wish to examine the presence of experiential effects across a range of chess abilities and playing history. Using a players current rating, we collected data for 40 players in each of 3 separate rating cohorts: 1700–1900, 2000–2200 and 2300–2500. Between October 23–31, 2023, we randomly selected 40 players within each of these rating ranges that had recently played bullet games of any format, and scraped information about the games they had played up to that date. These rating cohorts correspond to a range of moderate to high chess ability.2Lichess provides a weekly distribution of ratings on the platform. At the time of writing, 47 % of bullet players had a rating lower than 1,500, while only 1.5 % had a rating higher than 2,500. We also collect all games played by 25 grandmaster (GM) accounts on Lichess in early 2024. Grandmaster is an official title awarded to players who demonstrate excellent ability in chess. At the time of writing, this extremely selective title has only been awarded to 2087 players in history. For the GMs we selected, the average rating was 3,055 for Bullet and 2,854 for Blitz games. We denote the 40 players in each rating cohort and the 25 GMs as “focal” players in this paper. We have made this data, along with the code used to scrape it, publicly available in our associated Github repository (https://github.com/OwenWard/Chess_Winner).

For every game by each focal player in the 60+0 and 180+0 time controls, we observe their current rating, the rating of their opponent, the colour of each player and the outcome of the game. Importantly, we note that we are unable to obtain the uncertainty in a player’s rating (known as the rating deviation), which is a key component in the Glicko-2 rating system. For brevity, we focus our findings on the 1700–1900 and GM cohorts in what follows. The results obtained for the other cohorts, unless noted, agree with those presented below, with some of the corresponding analyses included in the Appendix.

### 2.1 Data summary

We summarize the total number of games we use for both the 1700–1900 and GM cohort in Table 1. For the 1700–1900 cohort we have 36 focal players playing 60+0 Bullet Games. This is more than 87 % of all rated bullet games they play (across all possible time limits and increments), with each averaging over 16,000 games. For the grandmasters 60+0 games correspond to 75 % of all bullet games played, averaging over 8,000 per player. The corresponding statistics for 180+0 Blitz games are shown in Table 1. Bullet games are considerably more popular across all cohorts, perhaps due to the short time required.

**Table 1:** Summary statistics for bullet and blitz games by cohort and format. Here percent of games corresponds to how many of all bullet/blitz games were of this specific time format.

Game type	Cohort	Num. players	Games (count)	Percent of games	Avg games per player
Bullet (60+0)	1700–1900	36	590,253	87 %	16,396
Bullet (60+0)	Grandmasters	23	196,522	75 %	8,544
Blitz (180+0)	1700–1900	36	107,670	58 %	4,141
Blitz (180+0)	Grandmasters	23	34,251	98 %	1,489

#### 2.1.1 The time scale of experiential effects

A key question in the study of experiential effects is if and how these effects persist over time. In the context of teams sports such as football, there may be several days between subsequent games, which could interfere with such effects. Due to the short time format most popular in online chess, it is common for players to play many games in quick succession. For bullet and blitz games where the players do not receive an increment, the maximum game length is simply the sum of the time both players start with.

**Figure 2:**
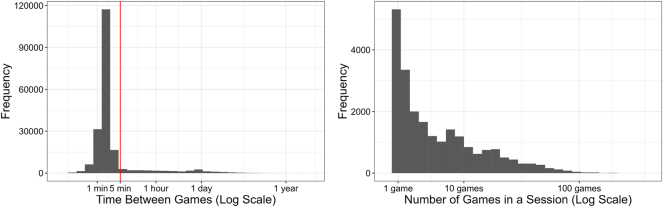
Time gaps between successive bullet games (left) and the distribution of the lengths of a session of bullet games (right) for the GM cohort.

In Figure 2(left) we show the time between the start of successive bullet games in the GM cohort on a log scale. The maximum length of these games is 2 min. Across all grandmaster games, 88.6 % of the games are played within 5 min of the previous game. This indicates that periods where players play more than one game in a row are very common (for the 1700–1900 cohort this is 85 % of all bullet games).

Based on this information we define bullet games played in the same session as those which start within 5 min of the start of the previous game. With this definition we can then determine how many games are played in a given session. We plot the distribution of the number of games in a session, from the GM cohort, in Figure 2 (right). The median number of games played in such a session is 4, with 76.3 % of all sessions involving more than 1 game. It is very common for an individual to play multiple games in quick succession. It is commonly thought that experiential effects are short term, with the result of the previous competition having an impact on the next (Franz et al. 2015). If experiential effects do occur, it seems plausible that they would be detected in games of this form, where players who win or lose can almost immediately enter another contest.

While it is believed that winner-loser effects occur based on the most recent history, we wish to investigate if longer term effects can exist also. In the model described below we will investigate the presence of these effects based on the result immediately proceeding the current game only. However, we will also investigate if these effects have a longer duration by using the performance in the previous 10 games in a session as a predictor of the current outcome. It is still relatively common for players to play 10 or more games in succession, with 26.3 % of sessions played by GMs, as we have defined them, containing more than 10 games.3We see similar proportions for the 1700–1900 cohort. Fitting our proposed model taking account of this longer history allows investigation of more prolonged experiential effects.

## 3 Modeling winner-loser effects with a hierarchical logistic regression

We have large volumes of games where players are randomly assigned to opponents of similar ability and we observe potential confounding factors such as this difference in ability and the colour each player plays. This makes this data particularly well suited to investigate the role of experiential effects on game results.

To investigate these effects, we model the outcome of each game played by the focal players in our data using a Bayesian hierarchical logistic regression model. The model we consider is general and can readily be applied to many forms of data consisting of repeated competition over time, identifying both individual and population level effects in such data. The Bayesian nature of our model provides natural uncertainty quantification in our estimates along with tools for checking the validity of our model.

The probability a focal player wins an individual game is given by

(1) $$P\left(y_{ij}=1\right)=p_{ij}=\frac{1}{1+\exp\left(-\left(\alpha_{j}+\beta_{j}{\tilde{x}}_{ij}^{n}+\gamma^{T}z_{ij}\right)\right)}.$$

Here *p*_*ij*_ is the probability of the *j*-th focal player winning their *i*-th game, *y*_*ij*_. *α*_*j*_ is a player level random effect, indicating a player’s individual ability to win a game against an equally rated opponent when playing with the black pieces. *β*_*j*_ is a player level random effect, accounting for the win ratio ${\tilde{x}}_{ij}^{n}$ of the focal player over their previous *n* games. We discuss this in more detail below. ***γ*** is a vector of fixed effects corresponding to two individual game covariates, the colour played by the focal player and the difference in rating between the focal player and their opponent. We partially pool the *α* and *β* coefficients with a bivariate normal prior distribution and place independent normal priors on the fixed effects. The prior specification for all parameters is given by

(2) $$\left(\begin{array}{c} \alpha_{j}\\ \beta_{j} \end{array}\right)∼N\left(\mu ,\Sigma \right),$$

and

$$\gamma_{1}∼N\left(0,\sigma_{g_{1}}^{2}\right),\;\gamma_{2}∼N\left(0,\sigma_{g_{2}}^{2}\right)$$

where we have

(3) $$\mu =\left(\begin{array}{c} 0\\ \mu_{\beta } \end{array}\right)\text{ and }\Sigma =\left(\begin{array}{cc} \tau_{1} & 0\\ 0 & \tau_{2} \end{array}\right)\Omega \left(\begin{array}{cc} \tau_{1} & 0\\ 0 & \tau_{2} \end{array}\right).$$

We then place independent hyperpriors on each of these parameters, with

$$\mu_{\beta }∼N\left(0,\sigma^{2}\right),$$

$$\tau_{1},\tau_{2},\sigma ,\sigma_{g_{1}},\sigma_{g_{2}}∼N_{+}\left(0,1\right)$$

and

Ω∼LKJcorr(2),

where LKJcorr(2) is a Lewandowski-Kurowicka-Joe (LKJ) distribution with shape parameter 2 (Lewandowski et al. 2009) and $N_{+}\left(0,1\right)$ is a positively truncated standard normal distribution.

As we are interested in identifying potential global winner-loser effects, we place a prior on *μ*_*β*_, the global winner-loser effect. The aim of inferring this parameter is to identify if there is evidence for a common effect across all players, rather than isolated to individual players. *μ*_*β*_ > 0 would indicate the presence of a cohort level winner-loser effect. If the focal player had won (lost) more recent games than their overall win proportion, this would lead to their probability of winning the next game increasing (decreasing). *μ*_*β*_ < 0 would indicate a reverse relationship, where poor recent performance would lead to an increased win probability in future games. We note that this model gives equal weight to both these winner and loser effects and cannot identify them separately, an important natural extension. This model can then be fit for various choices of *n*, the number of previous games considered in ${\tilde{x}}_{ij}^{n}$ . As discussed above, we will consider *n* = 1 and *n* = 10, investigating both immediate and longer term experiential effects. In the Appendix we demonstrate similar results with *n* = 5 also.

We normalize the win ratio by the cumulative win ratio of player *j* up to the *i*-th game. If $x_{ij}^{n}$ is the proportion of games the *j*-th player has won in the past *n* games then ${\tilde{x}}_{ij}^{n}=x_{ij}^{n}-{\bar{x}}_{j}$ , where ${\bar{x}}_{j}$ is their cumulative win proportion up to the current game. Importantly, we consider previous games to only be those played in the current session, as defined previously. If this is the first game in a session then we define ${\tilde{x}}_{ij}^{n}=0$ . Note that if *n* = 1, meaning we only consider the previous game in the session, then ${\tilde{x}}_{ij}^{n}$ will be positive if the previous game was a victory, and negative if the previous game was a loss. We note that the reliability of this metric depends on *n*, and it will be calculated using only the previous *n* games in that session. The potential consequences of the reliability of this metric are discussed further in Section 6.

For ***z***_***ij***_ we incorporate 2 covariates, namely the colour played by the focal player in an individual game and the rating difference between the focal player and their opponent. ***γ*** = (*γ*_1_, *γ*_2_) where *γ*_1_ describes how the win probability changes in going from playing as black to playing as white. *γ*_1_ > 0 would indicate that against an equally rated opponent, there would be an increased probability of winning when playing as white compared to playing as black. *γ*_2_ describes the relationship between win probability and the difference in rating between the focal player and their current opponent. *γ*_2_ > 0 would indicate that the more highly rated a focal player is than their opponent, the higher the probability they would win a match, accounting for the colour assigned to each player.

We decompose the covariance matrix Σ into a correlation matrix Ω and the individual scale of the two random effects. For the correlation matrix we choose a Lewandowski-Kurowicka-Joe (LKJ) distribution as the prior, with shape parameter 2 (Lewandowski et al. 2009). This is a prior over the set of correlation matrices with the chosen shape parameter favoring less correlation. We use independent half standard normal priors for the scale parameters *τ*_1_, *τ*_2_. In initial model fitting we found that less informative priors for the scale parameters, such as Inverse-Gamma, gave equivalent results while making posterior convergence more challenging.

This model is a natural approach to investigating this phenomenon in online chess, and builds on models which have been considered in both the sport analytics and animal behaviour literature. Franz et al. (2015) examine experiential effects in primates by modifying the ELO rating system. They modify the K-factor in this model, estimating this as a function of contest level predictors. This model could be applied to similar scenarios to the one we consider, however the ELO rating has largely been superseded by the Glicko-2 method. Our Bayesian approach also provides additional uncertainty quantification for identifying experiential effects. The model for hockey goalie performance of Ding et al. (2025) is similar to our approach, in that they fit a hierarchical logistic regression to contest outcomes and include historic performance as a predictor. Our model expands on this approach by considered a more flexible modeling structure, specifically designed to identify a global experiential effect. We also develop novel model checking methods. The flexibility of our model also allows it to be applied to a range of potential datasets consisting of repeated competition observed for a number of individuals.

## 4 The fitted model

We fit the proposed Bayesian model with Stan (Stan Development Team 2024), using the CmdStanR (Gabry et al. 2025) interface in R (R Core Team 2024). We use default settings for fitting these models, running 4 chains each with 1,000 warmup and 1,000 sampling iterations. We fit the proposed model for each of the four cohorts discussed previously. In each case the models indicate posterior convergence, as indicated by $\hat{R}$ values close to 1 and large effective sample sizes (Gelman et al. 2013). We discuss the results of these models for two cohorts: the 1700–1900 cohort and the GM cohort. Similar results for the other cohorts are included in the Appendix. All code required to reproduce this analysis will be available in the associated Github repository, along with access to the data used.

### 4.1 Comparing the fitted model for players of different ability

**Figure 3:**
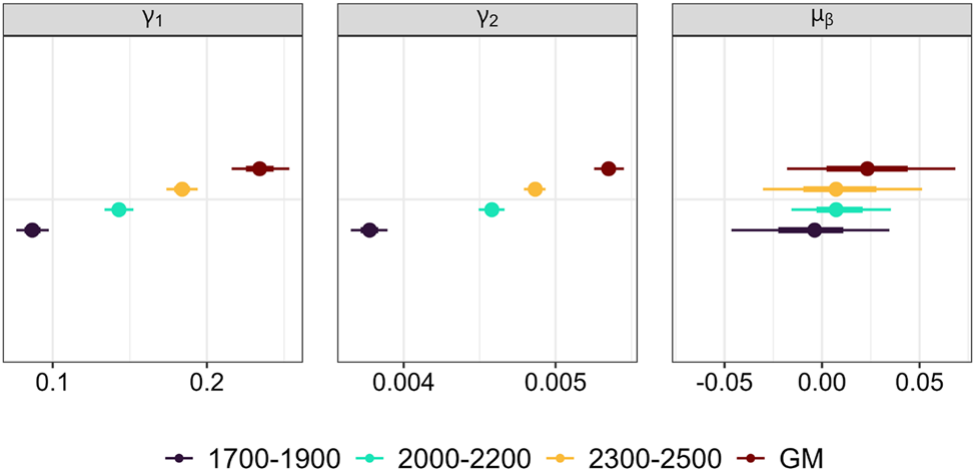
The 66 % and 95 % credible intervals of the estimated colour (*γ*_1_), rating (*γ*_2_) and global experiential (*μ*_*β*_) effects in the bullet games. The posterior means of the colour and rating effects increase with skill. There is little evidence for a global winner effect in any cohort.

We first fit the proposed model for standard bullet games as described above with *n* = 1, utilising the previous game in the same session as the only history. We examine the key global parameters (*γ*_1_, *γ*_2_, *μ*_*β*_), in all four cohorts, with posterior credible intervals for each shown in Figure 3. The main difference is seen in the fixed effects *γ*_1_ and *γ*_2_. The estimated posterior mean of the colour effect (*γ*_1_) is 0.087 and 0.23 for the 1700–1900 and GM cohorts respectively. For a player with a win probability of 0.5 playing as black against an equally rated opponent, playing as white would increase the win probability on average to 0.56 for the GMs but only 0.52 for the 1700–1900s. This agrees with common beliefs for the first move advantage for GMs. Among decisive results in top GM games, white wins 54–56 % of games, compared to black winning 44–46 % of games. It is also commonly believed that this advantage diminishes at lower levels of play (Kaufman 2021). Similarly, the estimated posterior mean of the rating effect (*γ*_2_) is 0.0038 and 0.0053 for the 1700–1900 and GM cohorts respectively. For a player with a win probability of 0.5 playing as black against an equally rated opponent, playing against an opponent rated 100 points lower increases the win probability on average to 0.63 for GMs and 0.59 for 1700–1900s. The increased impact of these effects in higher rated players seems reasonable, with elite players better able to capitalize on these advantages.

In the 1700–1900 cohort the posterior mean value for *μ*_*β*_ is centered around 0, indicating that there is little evidence for a global experiential effect. The posterior mean estimate for the GM cohort is positive, although the 95 % interval contains 0, indicating limited weak evidence for a positive experiential effects. We also include the estimates for each of these parameters for the two intermediate cohorts in Figure 3. We see a clear linear pattern in the estimates of *γ*_1_ and *γ*_2_, corresponding to increasing ability in the cohort. However, for all cohorts, the estimate of *μ*_*β*_ is centered close to 0.

**Figure 4:**
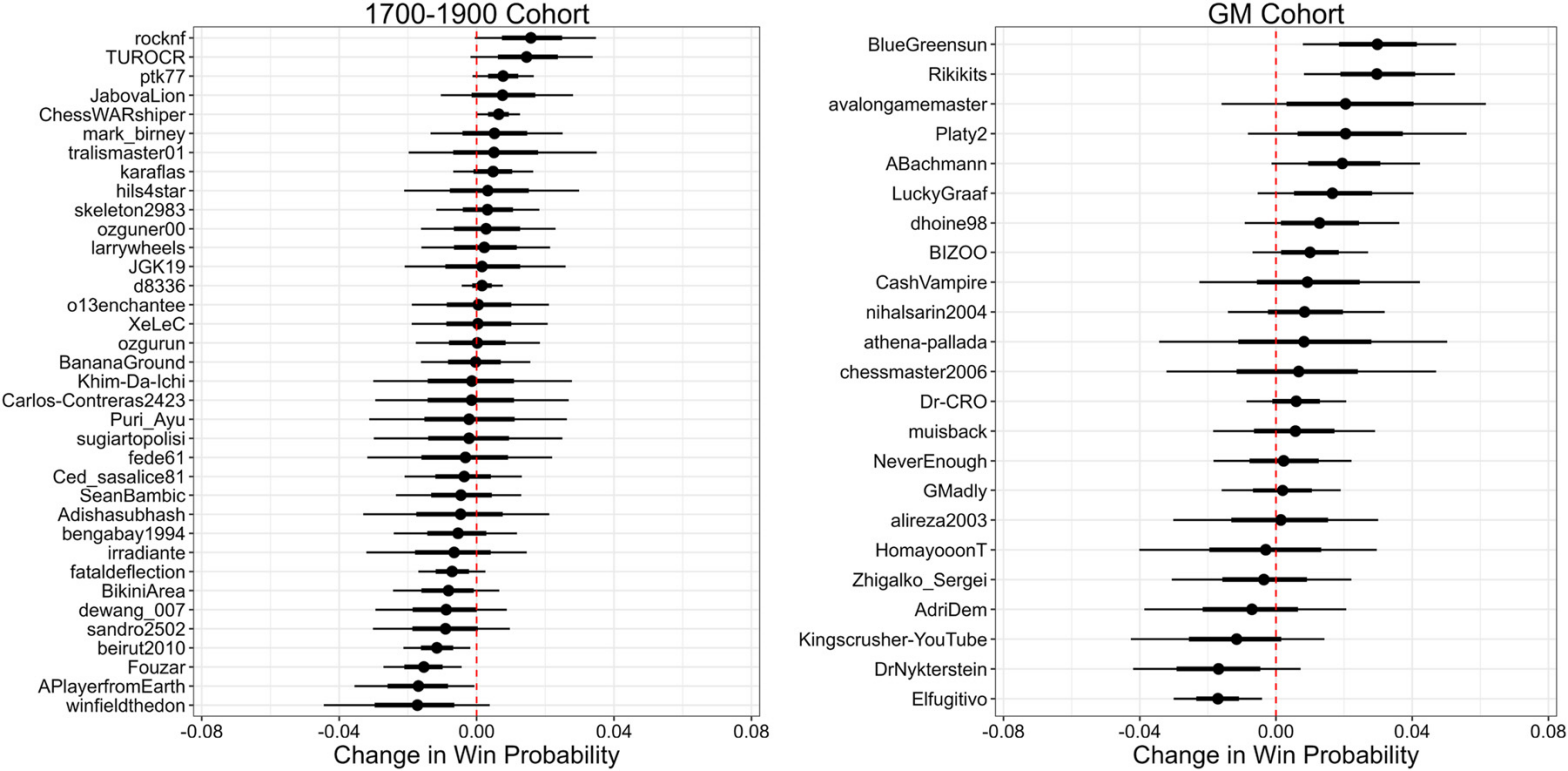
Interpretation of the estimated player-level experiential effects (*β*_*j*_ for player *j*) for the 1700–1900 cohort (left) and GM cohort (right). The change in win probability represents the additive boost in win probability a player receives from the experiential effect when changing their previous game from a loss to a win. The 66 % and 95 % credible intervals are shown. Most players exhibit little evidence for non-zero experiential effects, which results in changes in the win probability that are close to zero.

While we see little evidence for a global winner-/loser-effect, we also wish to examine the individual experiential effects and how these vary across focal players. In Figure 4 we take the posterior estimates of *β*_*j*_ for each player and transform them into the change in predicted win probability. In particular, if all other parameters were fixed, we compute how much the win probability would increase in going from losing the previous game to winning it.

We show credible intervals of the posterior estimates of the change in win probability for 36 focal players in the 1700–1900 cohort4Four players in the 1700–1900 cohort were excluded due to not playing any 60+0 bullet games. and 25 in the GM cohort. We see that many of these players have changes close to 0. The posterior means for this quantity across these two cohorts range from −0.02 to 0.03. The majority of 95 % posterior intervals contain 0 (or are close to 0). These effects are small, and indicate small changes in the win probability based on performance in the previous game. One interesting observation here is that the player with the second smallest estimated change in the GM cohort, the username DrNykerstein, is one of the accounts of former world champion Magnus Carlsen. While this result seems to indicate that Carlsen is less likely to win the next game if he had won the previous game than other players, there are several potential factors which could explain this. One possible explanation concerns data quality. Carlsen is widely known for playing in an “erratic” manner in casual games online, and as seen in live streams, may not be taking such games seriously. In terms of the model used, it is possible that Carlsen is different to other players and (for example) has a winner and loser effect of different sizes. This would not be captured by our current model.

### 4.2 The use of different history

**Figure 5:**
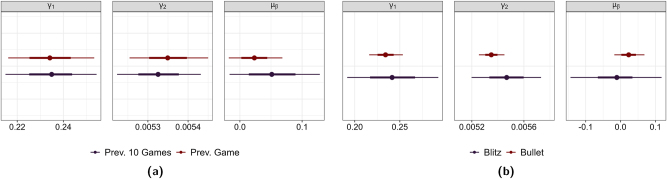
Comparison of the estimated fixed and global effects in varying settings for the GM cohort. In each case we show 66 % and 95 % posterior intervals. (a) Comparing the estimated parameters when *n* = 1 versus *n* = 10. (b) Comparing the estimated parameters using bullet or blitz data.

Our proposed model shows little evidence for winner-loser effects above when *n* = 1, which corresponds to the result of the previous game alone impacting subsequent performance. However, it is possible that experiential effects could require longer time periods to develop. To investigate this we refit the above model now using *n* = 10 for computing the win ratio ${\tilde{x}}_{ij}^{n}$ . It is relatively common for sessions involving more than 10 games to be played consecutively, with more than 20 % of all sessions across both cohorts containing at least 11 games. In Figure 5a we show the posterior estimates for the three key model parameters for both *n* = 1 and *n* = 10 for the GM cohort. We note that the posterior distributions for all parameters except for *τ*_2_ are essentially identical with slightly more uncertainty with *n* = 10, due to the decreased number of sessions. The posterior mean estimate for *μ*_*β*_ is closer to 0 when *n* = 10, although the intervals show substantial overlap. The posterior distribution of *τ*_2_ is shifted towards larger values due to the increased variability of ${\tilde{x}}_{ij}^{n}$ when *n* = 10. Individual winner-loser effects (shown in the Appendix) show similar patterns with increased variability when *n* = 10, with a similar proportion of posterior intervals containing 0 in both cases. It does not appear that a longer term history provides additional evidence for the presence of strong or persistent experiential effects.

### 4.3 Comparing blitz and bullet games

The previous analysis has investigated the presence of experiential effects in short bullet games lasting at most 2 min. To consider the possibility that longer competition is required for such effects to form, we repeat the previous analysis now using blitz games. These are games of up to 6 min in length. Although these games are longer, it is still common for successive sequences of games. We define a focal player starting another blitz game within 7 min of their previous game as games played in the same session. With this definition, two thirds of sessions played in the GM cohort contain more than one game, along with almost three quarters of games in the 1700–1900 cohort. As described in Section 2, we have substantially more bullet than blitz games for all players.

We compare the fit of our proposed model with *n* = 1 for the GM cohort. The posterior estimates of key parameters are shown in Figure 5b. The estimated fixed effects *γ*_1_, *γ*_2_ show larger variability in the smaller blitz dataset. In both cases, we see the distribution of the estimated global winner-/loser-effect is close to zero, with significantly more variability in the smaller Blitz dataset.

**Figure 6:**
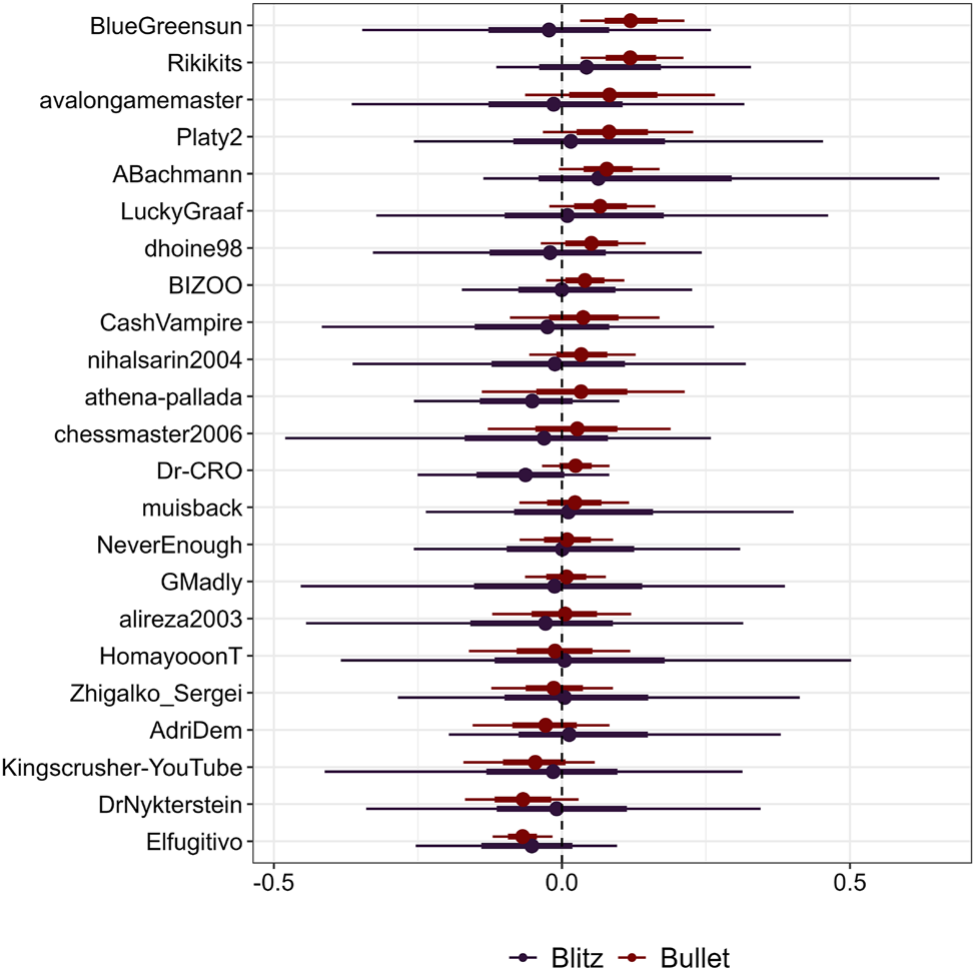
Winner-loser effects for the GM data, from fitting the proposed model to bullet and blitz data. We see considerably larger variability with the smaller blitz dataset.

In Figure 6 we show the estimated individual winner-loser effects for the members of the GM cohort using both the blitz and bullet data. Some of the distributions appear to shift slightly with the blitz data but all demonstrate larger variability using the blitz data. This is expected, as all play more bullet games (on average 9,000 bullet games compared to 1,300 blitz games in this data, with an ever bigger difference in other cohorts).

Overall, we see little evidence for consistent experiential effects from fitting the proposed Bayesian logistic regression model to large volumes of online chess data. Across a variety of formats and time scales for the formation of these effects, we do not see any evidence for such effects across the population of players considered. While there is some evidence for individual players displaying winner-loser effects, and some evidence of a small global effect in the highest rated players, these effects appear to be weak. An important concern with the current analysis, investigating these effects in the context of recent performance, is the potential for high temporal correlation between successive games. In Section 5 we consider this and provide several tools to validate our model.

## 5 Model validation

The previous sections demonstrated that, under the proposed hierarchical regression model, there is little evidence for a consistent winner-/loser-effect across the population of online chess players studied. However, our model is very much a simplification of the complex nature of such data. One important consideration which our model does not account for is the temporal nature of this data. Many of the focal players in these datasets have several years of games in the dataset. It is possible that potential experiential effects can change over time, which we would fail to capture in this model. There is the potential for strong temporal dependence in this data which we do not account for in our simplified model. Below we consider one method to evaluate the fit of our proposed model in the context of these potential issues. We include an additional model checking procedure in the Appendix, examining permutation distributions of the game results. We also include there a simulation study, indicating roughly how many games would be required to detect experiential effects of varying sizes. We demonstrate that while our model may somewhat suffer from ignoring the clear temporal structure in this data, the current model can capture much of the dependence in this data.

### 5.1 Posterior predictive checks

Given the Bayesian model used here, a natural tool to assess our proposed model is to utilise posterior predictive checking. This is a widely used procedure where replicated data under the fitted Bayesian model is compared to the original observed data (Gelman et al. 2013). Following the notation of Gelman et al. 2013 we define the posterior predictive distribution as

(4) $$p\left(y^{\text{rep}}|y\right)=\int p\left(y^{\text{rep}}|\theta \right)p\left(\theta |y\right)d\theta ,$$

where *θ* is the vector of parameters in the model. We can then draw replicated data from this distribution, namely we can simulate the result of a given match, given the covariates and each posterior draw of the model parameters. A failure of these replications to match the observed data can identify potential flaws in the model. These posterior predictive checks are often done using some generated quantity from the original dataset. Here, a natural quantity to consider is the number of games won by a focal player in the observed data. An extension of this is to consider the generated quantity corresponding to the rating evolution of a player over replicated datasets, simulating each game result and then using these outcomes to construct the game history for future games. This is the quantity we will use for posterior predictive checking below.

Given the size of our original dataset, creating thousands of posterior predictive replications of each game has large memory requirements. Instead, we will restrict our data to a selection of focal players in the GM cohort. For these players we utilise their 2,000 most recent bullet games in the dataset. We will use the first half of this data to estimate the posterior distribution, before then simulating the results of the games in the second half, using each of the draws from the posterior and the value of ${\tilde{x}}_{ij}^{n}$ based on the simulated result of the previous game with this set of posterior draws. For each set of parameter draws from the posterior we can then compute the estimated ranking under a Glicko-2 rating system, similar to that used by Lichess. We use default values for the constant parameters in this model, which gives similar results to the (unknown) parameters used by Lichess. This gives us posterior predictive draws of the rating of a player over 1,000 games, which we can then compare to the true evolution of these scores.

**Figure 7:**
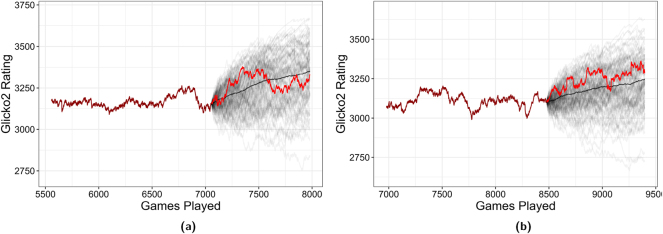
Posterior predictive distribution of Glicko-2 ratings for 2 GMs. We show the true Lichess rating in red, with 4,000 draws of the predicted rating evolution. The mean of these draws is shown in the solid black line. (a) Posterior predictive distribution for Magnus Carlsen. (b) Posterior predictive distribution for Nihal Sarin.

We fit our previous proposed model with *n* = 1 on the previous 1,000 games before then constructing a posterior predictive distribution of the Glicko-2 rating of each player using the final 1,000 games for the GM cohort. As we use the standard number of chains (4) and sampling iterarions (1,000) in CmdStanR, this gives us 4,000 posterior draws for each parameter. We then use each set of draws to simulate the results of each game in the last 1,000 games. This gives us 4,000 replications of the 1,000 games played by each focal player and the corresponding evolution of their Glicko-2 score. We show these replications for 2 GMs in Figure 7. While these replications show large uncertainty we see that they capture the overall trend well, in particular capturing the steady increase in rating shown in Figure 7b. This indicates that our model is able to predict the future temporal evolution of the ability of players well. We include an additional model checking approach, examining the results of our model when we permute the game results in the Appendix, along with this posterior predictive check for players from each of the other cohorts.

## 6 Summary, future directions

In this work we have proposed a Bayesian hierarchical logistic regression model to investigate the presence of experiential effects in online chess. Having fit this model to cohorts of players of varying ability we find that there is no strong evidence for the presence of global winner/loser-effects, as commonly defined in the literature. However, a small proportion of individual players from all cohorts show evidence of possessing such effects. This is consistent across multiple formats of chess and the number of previous games that this effect could persist over. Our model is a general Bayesian model which can easily be modified to other data of this form, providing a tool for estimating both individual and population level experiential effects. While we do not believe that our model perfectly describes the dynamics of chess, our model checking procedures indicate that our model appears reasonable and captures many of the key characteristics of this data. Our results indicate that a more flexible model may be required for players of higher ability, such as GMs, where we saw some limited evidence for *μ*_*β*_ > 0. This would need to be investigated further, and could be related to the larger impact of the covariates (color and rating difference) in games between better players. Another concern which arises when examining these top players is that the opponent pool is significantly smaller. As such, the assumptions of playing random opponents may not be appropriate for particularly strong players. Modifying our approach to account for non-random opponents is an important future extension, along with examining the reliability of the history covariate we use here. Our inference of experiential effects rely on $x_{ij}^{n}$ , the number of games used to measure recent performance, which can be estimated for different choices of *n*. Given we saw similar results when fitting our model with *n* = 1 and *n* = 10 (along with showing *n* = 5 in the Appendix), we do not believe that the choice of this covariate impacts our results. However, this choice of $x_{ij}^{n}$ should be investigated further and could potentially be improved. Similarly, a key advancement of the Glicko-2 rating system is the ability to incorporate individual rating deviation when examining the rating difference between players Glickman (2012). This gives a more reliable measure of the difference in ability between opponents, however this rating deviation is not available from the Lichess API. Being able to obtain and use this deviation could significantly improve our model.

There are many other potential future directions which could expand on this work. In shorter chess games it is common to attempt to “flag” your opponent, aiming to beat them by forcing them to run out of time. A large proportion of bullet games are won this way (24 % of bullet games in the GM cohort) and results of this form may have a different impact on future performance than other endings. We have also not attempted to separate out winner and loser effects or allow them to have different magnitudes in the current formulation. The current model also does not consider draws (which are rare in this data) and the potential impact they could have on future performance. Similarly, while we consider Bullet and Blitz games separately here we could also use both for predicting the outcome of either format, although it is very uncommon for players to switch between formats in a given session. More broadly, given the general nature of the model proposed here, we hope to also apply it to recent large animal interaction datasets, where the presence of experiential effects remains hotly debated.

## Figures and Tables

**Figure A1:**
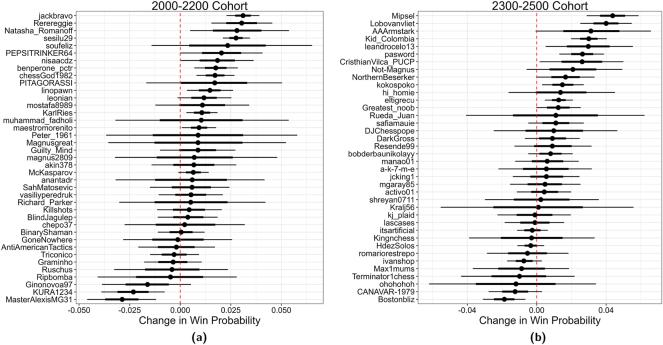
The estimated change in win probability for the other two cohorts we consider. (a) Players in the 2000–2200 cohort. (b) Players in the 2300–2500 cohort.

**Figure A2:**
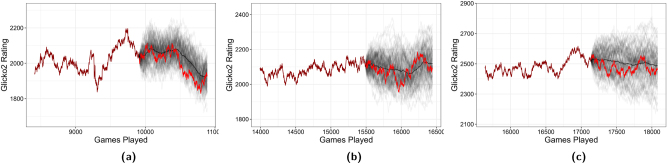
Posterior predictive distribution of Glicko-2 ratings for 3 players in different rating cohorts. We show the true Lichess rating in red, with 4,000 draws of the predicted rating evolution. The mean of these draws is shown in the solid black line. (a) Posterior predictive distribution for “APlayerfromEarth” (1700–1900 cohort). (b) Posterior predictive distribution for “Rerereggie” (2000–2200 cohort). (c) Posterior predictive distribution for “Lobovanvliet” (2,300–2,500 cohort).

**Figure A3:**
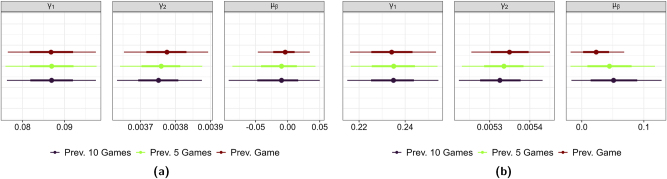
Posterior estimates for the key global parameters *γ*_1_, *γ*_2_, *μ*_*β*_ as we vary *n* in our fitted model. (a) 1700–1900 cohort. (b) GM cohort.

**Figure A4:**
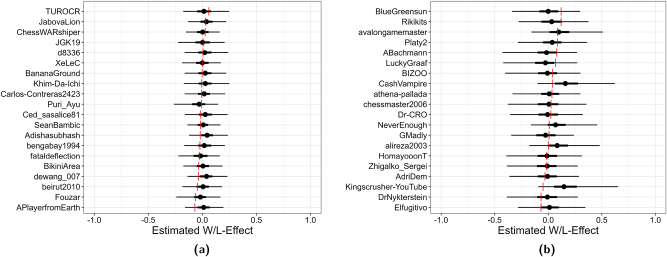
Posterior mean estimates for winner/loser-effects after permuting the game data. For each the true posterior mean estimate is shown in red, with the intervals representing 66 % and 95 % of the estimates obtained under the permutation distributions. (a) Permutation estimates for 20 players in 1700–1900 cohort. (b) Permutation estimates for 20 players in the GM cohort.

**Figure A5:**
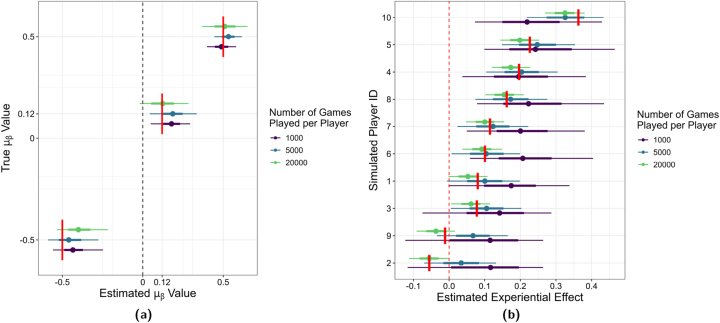
66 % and 95 % credible intervals for estimated effects for *μ*_*β*_ and *β*_*j*_. In both figures the red vertical lines represent the true values of each respective parameter. (a) The estimated values of *μ*_*β*_ for 3 different known effect sizes. (b) The estimated experiential effects for 10 simulated players for known *μ*_*β*_ = 0.12.

**Figure A6:**
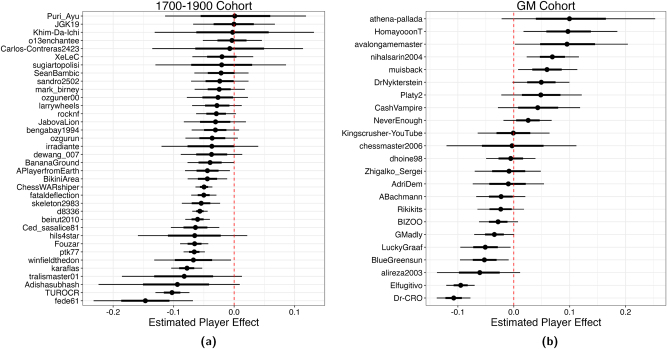
Estimated player effects for the 1700–1900 and GM cohorts. (a) Player effects for the 1700–1900 cohort. (b) Player effects for the GM cohort.

## Appendix: Additional analyses

Here we include some additional model fitting results which were omitted from the main text. In particular, we show selected model estimates and model diagnostics for the other cohorts considered along with an additional model checking procedure and simulation study. We also investigate the effect of using varying numbers of games for the historic influence.

### A.1 Examining winner-/loser-effects across cohorts

In Figure A1 we show the posterior distributions of individual *β* parameters for each player in the 2000–2200 and 2300–2500 cohorts. In each case we see similar results to those shown in the main text. The majority of posterior distributions for *β*_*j*_ contain zero, with a small number of players showing positive or negative estimates. This behaviour is common across all ranges of rating we considered.

### A.2 Posterior predictive checking for other cohorts

In Figure A2 we demonstrate further posterior predictive checks using players from cohorts not included in the main text. We again construct the posterior predictive distributions of the Glicko-2 rating evolution. We show sample players in the 1700–1900, 2000–2200 and 2300–2500 cohorts. In each case we see that the predictive distribution appears to match the general pattern over future games, indicating our model is able to predict the temporal nature of the ranking evolution.

### A.3 Investigating the role of *n*

In the main text we fit our model with *n* = 1 and *n* = 10, considering the previous performance over the previous game and the previous 10 games respectively. Given that many playing sessions contain less than 10 games, a natural question is whether *n* = 10 is too long a time scale for potential experiential effects to persist. In Figure A3 we show the key global parameters *γ*_1_, *γ*_2_, *μ*_*β*_ for the 1700–1900 and GM cohorts, for *n* = 1, 5, 10. In each case we see that the estimates of these parameters are essentially identical, which is also the case for the individual parameters *β*_*j*_.

### A.4 Permuting the game results

As an alternative approach to assessing the robustness of our model, we consider permuting the data. A similar approach was considered by Ding et al. (2025), to examine evidence for a hot hand effect in hockey. For computational considerations, we perform this analysis with a subset of the complete bullet data. We use 20 players from each of the 1700–1900 and GM cohorts, taking the final 1,000 games from each. We randomly permute the results within each focal player’s data 1,000 times, giving us 1,000 permuted datasets. For each of these we fit our proposed model with *n* = 1. We then examine the distribution of the posterior means of the experiential effects that are generated from each of these model fits.

Figure A4 shows these “permutation distributions” for 20 players in the 1700–1900 cohort and 20 players in the GM cohort, with the central 66 % and 95 % intervals for each estimate of *β*_*j*_ under these permutation distributions. For all players the true estimated experiential effects lie within these 95 % permutation intervals. This aligns with our previous results, indicating that individual experiential effects are small and close to zero. We note that when we repeat this analysis for the fixed effects *γ*_1_ and *γ*_2_ the distribution of these effects under the permutations is clearly different from the estimated effects, for all cohorts, as would be expected.

### A.5 Simulation study

Our previous analyses reveal that the experiential effects that we are interested in, if they exist, are likely small. In this case, even the large number of games analysed here may not be sufficient to detect such small effects. To investigate this further we construct a simulation study, examining when these effects can be identified from a cohort of 10 simulated focal players. To do this, we simulate game outcomes from the model proposed in Section 3. The values of the parameters in this model are given by:

– *γ*_1_ = 0.091
– *γ*_2_ = 0.0038
– *α*_*j*_ ∼ *N*(−0.075, 0.025). This distribution approximates the estimates from Figure A6a.
– *β*_*j*_ ∼ *N*(*μ*_*β*_, 0.01). This variance is close to the posterior mean for *τ*_2_ estimated from the 1700–1900 cohort.
– The rating difference for any given game is sampled from a Laplace distribution with mean 0 and scale 30. This closely resembles the actual rating differences in the 1700–1900 cohort.
– For any given game, the simulated player had 0.5 probability of being white and 0.5 probability of being black.

We choose to only look at the previous game for history here (*n* = 1). To investigate what effects we can detect we vary the true value of *μ*_*β*_ in this simulation along with the number of games played by each player, using *μ*_*β*_ = (−0.5, 0.12, 0.5) and *n*_per player_ = (1,000, 5,000, 20,000) we chose two large global experiential effects in different directions and one small positive experiential effect, giving a range of possible true effects. Note that *μ*_*β*_ = 0.12 equates to an approximate average boost in win percentage by 3 % when coming from a win for all players in the dataset. We chose a maximum of 20,000 games per player to be somewhat larger than the average number of games per player in the 1700–1900 cohort. We show the posterior estimates of *μ*_*β*_ and *β*_*j*_ for each of these 9 simulation settings in Figure A5.

In each simulation study, the 95 % credible intervals contain the true parameter values for both *μ*_*β*_ and each *β*_*j*_. We note that when estimating individual *β*_*j*_, we need a large number of games to get posterior intervals which do not contain 0 when the true value is non-zero. While a large number of games is required to detect these small effects, as shown in our model checking procedures, these small effects have little importance in describing the outcomes of these games.

### A.6 Player effects

Finally, we show some of the estimated player effects which we have excluded from the main text. In Figure A6 we show the posterior estimates for each *α*_*j*_ for the 1700–1900 and GM cohort. For the lower ability players many of these effects appear to be negative. This agrees with intuition that these players are likely to win less than half of their games. For the GM cohort we see some strong positive player effects, including for Magnus Carlsen.
